# Advances in protein subunit vaccines against H1N1/09 influenza

**DOI:** 10.3389/fimmu.2024.1499754

**Published:** 2024-11-22

**Authors:** Yu Zhang, Jingyao Gao, Wenqi Xu, Xingyu Huo, Jingyan Wang, Yirui Xu, Wenting Ding, Zeliang Guo, Rongzeng Liu

**Affiliations:** ^1^ Department of Immunology, College of Basic Medicine and Forensic Medicine, Henan University of Science and Technology, Luoyang, China; ^2^ Department of Medical Imaging, School of Medicine, Zhoukou Vocational and Technical College, Zhoukou, China

**Keywords:** influenza vaccine, A/H1N1pdm09, yeast, adjuvant, animal model

## Abstract

The A/H1N1pdm09 influenza virus, which caused the 2009 pandemic, has since become a recurring strain in seasonal influenza outbreaks. Given the ongoing threat of influenza, protein subunit vaccines have garnered significant attention for their safety and effectiveness. This review seeks to highlight the latest developments in protein subunit vaccines that specifically target the A/H1N1pdm09 virus. It will also examine the structure and replication cycle of influenza A viruses and compare different types of influenza vaccines. Additionally, the review will address key aspects of H1N1 protein subunit vaccine development, such as antigen selection, protein expression systems, and the use of adjuvants. The role of animal models in evaluating these vaccines will also be discussed. Despite challenges like antigenic variability and the complexities of vaccine production and distribution, protein subunit vaccines remain a promising option for future influenza prevention efforts.

## 1 Introduction

Influenza is a major respiratory illness caused by influenza viruses, which are classified as enveloped single-stranded negative-sense RNA viruses (1). These viruses are divided into four genera, A, B, C, and D, based on the antigenic variations in the nucleoprotein (NP) and matrix 1 (M1) proteins (2). Influenza A viruses are particularly notable for their ability to infect a wide range of host species and their potential to cause pandemics through interspecies transmission (3). The classification of influenza A viruses is further determined by the antigenic variation of hemagglutinin (HA) and neuraminidase (NA), with 18 HA subtypes (H1-H18) and 11 NA subtypes (N1-N11) identified (4). The H1N1 subtype has played a critical role in several pandemics among the various seasonal influenza virus types. This paper will focus on protein subunit vaccines that target the A/H1N1pdm09 strain, responsible for the 2009 H1N1 pandemic.

Annual influenza outbreaks affect people of all ages, with those having pre-existing conditions like obesity, cardiovascular disease, diabetes, chronic respiratory illnesses, and kidney disease at higher risk for severe outcomes (5–9). Influenza A viruses infiltrate the human respiratory system via the nose, mouth or even the eyes, and they travel through the respiratory tract, seeking appropriate host cells (10). Symptoms associated with acute infection encompass cough, rhinorrhea, pharyngitis, fatigue and fever (11), which may subsequently escalate to acute respiratory distress syndrome or influenza-associated pneumonia, potentially leading to hospitalization and/or mortality due to respiratory failure (12, 13). Furthermore, infection with the pandemic H1N1 strain has been identified as a risk factor for the development of narcolepsy type 1 (14, 15). These findings highlight the importance of influenza vaccination in reducing the incidence of the disease and its complications (16).

**Figure 1:**
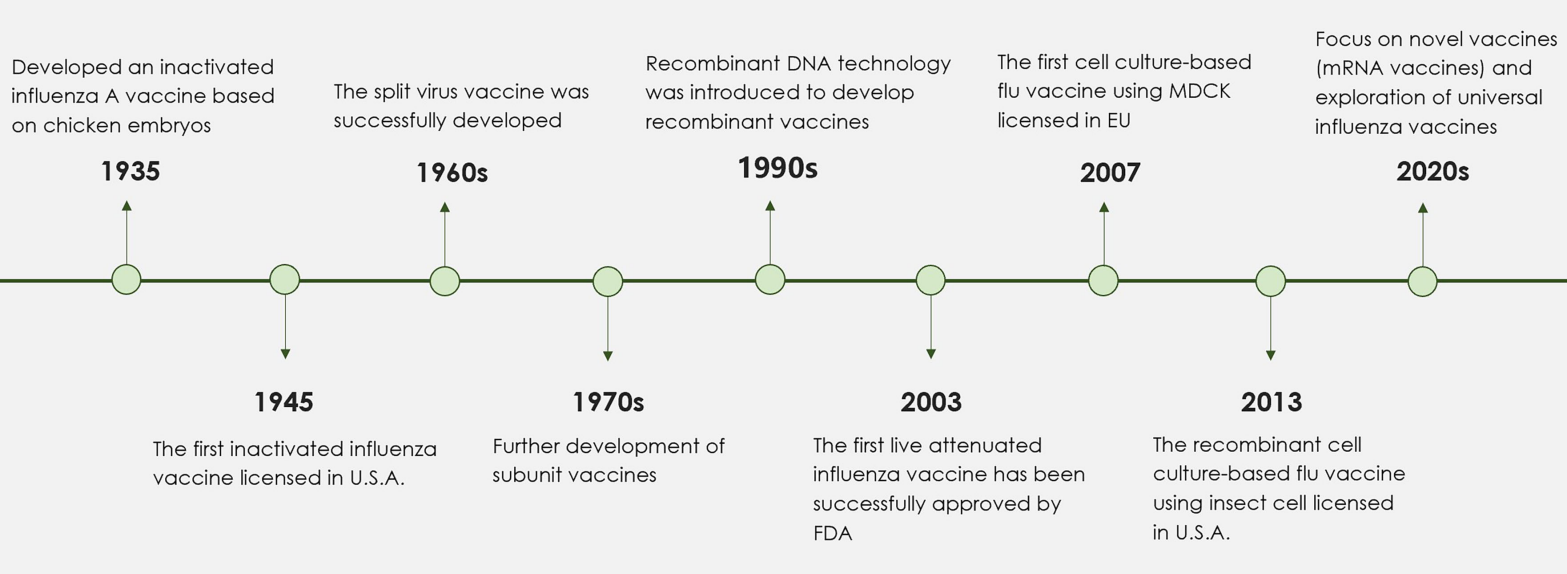
A timeline detailing the key stages in the development of influenza vaccines. Since the launch of the first inactivated influenza vaccine in 1945, significant progress in influenza vaccine technology has resulted in the development of split and subunit vaccines. In response to the challenges posed by egg-based influenza vaccines, successful alternatives utilizing cell culture methods have been established. The 1990s marked the introduction of adjuvants, including aluminum adjuvant and MF59, which notably enhanced the immunogenicity of influenza vaccines. More recently, the application of mRNA technology and nanoparticles has spurred the development of innovative influenza vaccines. Future research efforts are anticipated to focus on the formulation of broad-spectrum influenza vaccines designed to reduce the risks associated with potential pandemics.

Currently, there are three primary types of influenza vaccines used in humans, as shown in **Table 1**: inactivated influenza vaccines, live attenuated influenza vaccines, and recombinant subunit vaccines (17). The development process for these vaccines involves several critical stages, illustrated in **Figure 1**. The production process for eggs-based inactivated influenza vaccine is time-consuming, increasing the risk of viral mutations that may lead to a mismatch between the vaccine and circulating strains (18). On the other hand, live attenuated influenza vaccines are capable of eliciting both humoral and cellular immune response (19). However, there are safety concerns with live attenuated influenza vaccines due to the potential risk of the attenuated virus reverting to a pathogenic form (20). Meanwhile, research is ongoing into new H1N1 vaccines, including nucleic acid and viral-vector vaccines (21, 22). DNA vaccines offer advantages such as rapid design, efficient production, and improved thermostability, though long-term safety data are still lacking (23). Recombinant subunit vaccines, which use only specific viral proteins to elicit an immune response, are advantageous in terms of safety, as they exclude live viruses and can be produced efficiently at scale. In recent years, the trend has shifted toward subunit vaccine formulations that combine specific antigenic components with a potent adjuvant. This strategy ensures a well-defined antigen composition, reduces the risk of pathogenicity, and enables scalable, cost-effective antigen production and purification (24). At present, several protein subunit vaccines incorporating the A/H1N1pdm09 strain are undergoing clinical trials (**Table 2**).

**Table 1:** FDA-approved influenza vaccines (including the H1N1 pdm09 strain).

Production Platform	Adjuvant	Dose	Age	Tradename (Manufacturer)
Embryonated egg	None	15 µg	≥4 yrs	H1N1 2009 monovalent vaccine (Novartis)
Embryonated egg	None	15 µg	≥6 mo	H1N1 2009 monovalent vaccine (Sanofi Pasteur)
	None	36µg (9 µg HA/strain)	18-64 yrs	Fluzone (H1N1 component) (Sanofi Pasteur)
Baculovirus/Insect cell	None	135 µg (45 µg HA/strain)	≥18 yrs	Flublok (Sanofi Pasteur)
	None	180 µg (45 µg HA/strain)	≥18 yrs	Flublok Quadrivalent (Sanofi Pasteur)
Embryonated egg	MF59	45 µg (15 µg HA/strain)	≥65 yrs	FLUAD (Seqirus)
Embryonated egg	MF59	60 µg (15 µg HA/strain)	≥65 yrs	FLUAD Quadrivalent (Seqirus)
Embryonated egg	None	60 µg (15 µg HA/strain)	≥6 mo	Afluria Quadrivalent (Seqirus)
Madin-Darby canine kidney (MDCK) cell	None	60 µg (15 µg HA/strain)	≥6 mo	Flucelvax Quadrivalent (Seqirus)
Embryonated egg	None	45 µg (15 µg HA/strain)	≥4 yrs	Fluvirin (Seqirus)
Embryonated egg	None	45 µg (15 µg HA/strain)	≥18 yrs	Agriflu (Seqirus)
Embryonated egg	None	60 µg (15 µg HA/strain)	≥6 mo	Flulaval Quadrivalent (ID Biomedical Corporate of Quebec)
Embryonated egg	None	10^6.5-7.5^ FFU/0.2 ml dose	2-49 yrs	FluMist (MedImmune)

**Table 2:** Clinical trials of protein subunit influenza vaccines (including A/H1N1pdm09 strain).

Study Title	Study Design	Interventions	NCT Number	Phases	Sponsor
Study of the Safety and Immunogenicity of H1N1 Vaccine	Allocation: randomized**|** Intervention model: factorial assignment**|** Masking: single**|** Primary purpose: prevention	Biological: HAC1 Vaccine	NCT01177202	I	Fraunhofer, Center for Molecular Biotechnology
Evaluation of the Safety and Immunogenicity of a Recombinant Trivalent Nanoparticle Influenza Vaccine With Matrix M-1 Adjuvant (NanoFlu)	Allocation: randomized**|** Intervention model: parallel**|** Masking: triple**|** Primary purpose: prevention	Biological: NanoFlu Biological: Fluzone HD - Day 0/21 Other: Saline - Day 21	NCT03293498	I/ II	Novavax
Immunogenicity, Safety, and Tolerability of a Plant-Derived Seasonal Virus-Like-Particle Quadrivalent Influenza Vaccine in Adults	Allocation: randomized**|** Intervention model: parallel**|** Masking: quadruple**|** Primary purpose: prevention	Biological: Low/ Medium/ High dose of quadrivalent VLP vaccine Biological: Placebo	NCT02233816	II	Medicago
Immunogenicity, Safety and Tolerability of a Plant-Derived Seasonal Virus-Like Particles (VLP) Quadrivalent Influenza Vaccine in the Elderly Population	Allocation: randomized**|** Intervention model: parallel**|** Masking: quadruple**|** Primary purpose: prevention	Biological: Non-adjuvanted Low/ Medium/ High dose of quadrivalent VLP vaccine Biological: Adjuvanted Low/High dose of quadrivalent VLP vaccine Biological: Placebo	NCT02236052	II	Medicago
A Study to Prove Non-inferior Immunogenicity of Grippol Quadrivalent Compared to Grippol Plus	Allocation: randomized**|** Intervention model: parallel**|** Masking: quadruple**|** Primary purpose: prevention	Biological: Grippol Quadrivalent Biological: Grippol Plus	NCT06385821	III	NPO Petrovax
Pivotal, Multicenter, Observer-Blind, Randomized Study of Influenza A (H1N1)2009 Monovalent Subunit Vaccine With and Without Adjuvant in Children Ages 6 to <36 Months	Allocation: randomized**|** Intervention model: parallel**|** Masking: double**|** Primary purpose: prevention	Biological: MF59-eH1N1_f	NCT00996307	III	Novartis Vaccines
A Phase III, Open-Label, Single-Arm, Multicenter Study to Evaluate the Safety and Immunogenicity of a Trivalent, Surface Antigen Inactivated Subunit Influenza Virus Vaccine Produced in Mammalian Cell Culture (Optaflu®) in Healthy Adults	Allocation: N/A**|** Intervention model: single Group**|** Masking: none**|** Primary purpose: prevention	Biological: TIVc	NCT01880697	III	Novartis Vaccines
A Phase III, Randomized, Observer-Blind, Controlled, Multicenter Clinical Study to Evaluate the Efficacy, Safety and Immunogenicity of an MF59-Adjuvanted Quadrivalent Influenza Vaccine Compared to Non-influenza Vaccine Comparator in Adults ≥ 65 Years of Age	Allocation: randomized**|** Intervention model: parallel**|** Masking: triple**|** Primary purpose: prevention	Biological: aQIV Biological: Non-Influenza Comparator (Boostrix)	NCT02587221	III	Seqirus
A Phase 3, Randomized, Observer-blinded, Active-controlled Trial to Evaluate Immunogenicity & Safety of a Recombinant Quadrivalent Nanoparticle Influenza Vaccine With Matrix-M1™ Adjuvant Against Fluzone® Quadrivalent in Clinically Stable Adults ≥ 65 Years of Age	Allocation: randomized**|** Intervention model: parallel**|** Masking: quadruple**|** Primary purpose: prevention	Biological: NanoFlu Biological: Fluzone Quadrivalent	NCT04120194	III	Novavax
A Randomized, Observer-Blinded, Active-Controlled Study to Evaluate the Safety and Immunogenicity of a COVID-19 Influenza Combination Nanoparticle Vaccine and a Standalone Trivalent Nanoparticle Influenza Hemagglutinin Vaccine in Participants ≥ 65 Years of Age	Allocation: randomized**|** Intervention model: crossover**|** Masking: single**|** Primary purpose: prevention	Biological: CIC Vaccine Co-formulated tNIV2, SARSCoV-2 rS and Matrix-M Adjuvant Biological: Novavax COVID-19 Vaccine Biological: tNIV Vaccine Biological: Fluzone High Dose	NCT06291857	III	Novavax
A Clinical Study to Evaluate the Efficacy, Safety and Immunogenicity of an Adjuvanted Influenza Vaccine Compared to a Non adjuvanted Influenza Vaccine in Adults ≥65 Years of Age	Allocation: randomized**|** Intervention model: parallel**|** Masking: quadruple**|** Primary purpose: prevention	Biological: MF59-Adjuvanted Subunit Inactivated Influenza Vaccine Biological: Non adjuvanted Influenza Vaccine	NCT06087640	III/ III b	Seqirus

single: participant; double: participant, investigator; triple: participant, care provider, outcomes assessor; quadruple: participant, care provider, investigator, outcomes assessor.

The selection of antigen, expression system and adjuvant play a crucial role in determining the immunological effectiveness and coverage of a subunit vaccine. This review focuses on recent progress in developing protein subunit vaccines against the A/H1N1pdm09 virus. It includes an examination of the structural features and replication cycle of influenza A viruses, a comparison of different influenza vaccine types, and an evaluation of the protein expression systems and adjuvants used in H1N1 protein subunit vaccine formulations. Additionally, the review discusses the animal models used to assess the efficacy of these vaccines.

## 2 The structure and replication cycle of influenza A virus

**Figure 2:**
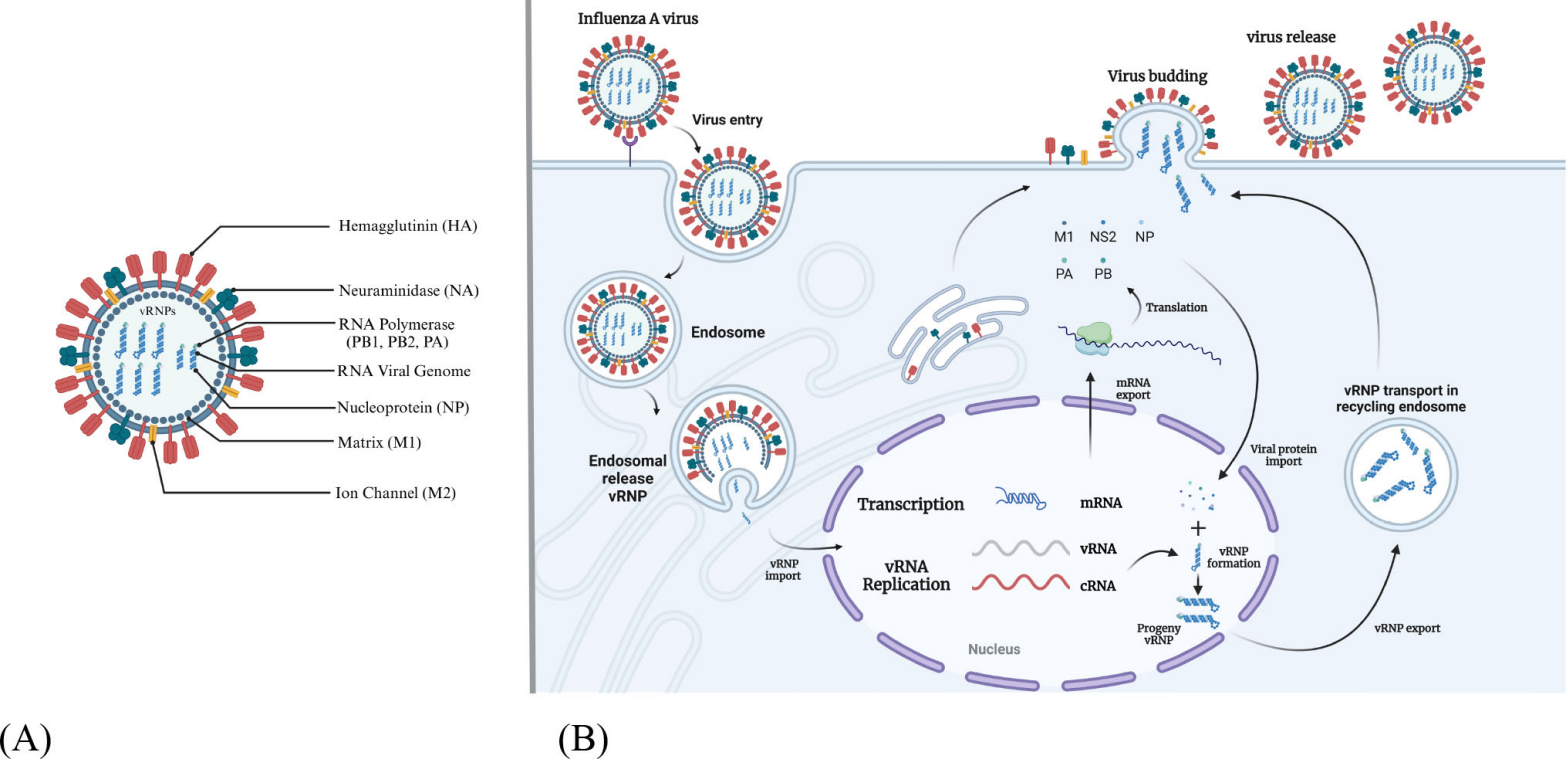
The structure and replication cycle of influenza A virus. **(A)** The structural composition of the influenza A virus is characterized by the presence of eleven distinct proteins. This includes three transmembrane proteins: HA, NA, and the M2 ion channel. Additionally, the inner surface of the envelope is comprised of the matrix protein M1, along with vRNPs, which consist of viral RNA, NP, and the polymerase complex proteins PB1, PB2 and PA. **(B)** The life cycle of the influenza A virus involves several key steps. Initially, the virus identifies sialic acid receptors through the HA-NA complex and enters host cells via the process of endocytosis. The acidic conditions within the endosome facilitate membrane fusion, resulting in the release of vRNP into the cytoplasm. The importin α/β1 complex then mediates the transport of vRNP to the nucleus, where RNA replication and transcription are completed with the assistance of viral polymerase. Following this, the progeny vRNP is exported back to the cytoplasm and directed to the budding membrane. The release of progeny viruses occurs through the interaction of HA-NA with sialic acid.

Influenza A viruses possess a genome that encodes a total of 11 proteins. These include the RNA polymerase subunit proteins (polymerase basic protein 1, polymerase basic protein 2, polymerase acid protein), HA, NA, NP, M1, membrane protein (M2) and nonstructural proteins NS1, NS2 and PB1-F2 (**Figure 2A**) (1). The core of influenza virus is the viral ribonucleoprotein complexes (vRNPs), which is the smallest functional unit of viral genome transcription and replication. The envelope of influenza virus consists of phospholipid bilayer derived from host cell membrane and two glycoproteins HA and NA. HA is the predominant component of influenza viruses and serves as a common antigenic element in vaccines (25). The production of HA precursor proteins occurs within the endoplasmic reticulum, followed by their cleavage into disulfide bonded HA1 and HA2 subunits, which collectively assemble into mature trimers, a process essential for viral infection. Each HA monomer is comprised of two structurally and functionally distinct domains: the head, primarily constituted by HA1, and the stem, predominantly made up of HA2 (26). Consequently, both HA1 and HA2 can be utilized as antigenic targets in the development of protein subunit influenza vaccines. NA is a tetramer glycoprotein, NA serves various functions within the replication cycle of the influenza virus, including aiding in the entry of the virus into host cells during the initial phase of infection and cleaving sialic acid to enhance the release of progeny viruses in subsequent stages (27).

Influenza A viruses initially attach to sialic acid receptors located on the surface of target cells through the HA protein, subsequently entering the host cells via endocytosis (**Figure 2B**) (3). The M2 ion channel facilitates the fusion of the viral envelope fuse with the endosomal membrane, resulting in the formation of maturing endosomes. The acidic environment within these maturing endosomes triggers the fusion of the viral and host endosomal membranes, thereby enabling the release of the viral ribonucleoproteins (vRNPs) into the cytoplasm (28). Certain cellular factors, including importin-α and importin-β1, are believed to play a role in the nuclear import of vRNPs (29). The vRNP functions as an autonomous unit that directs the synthesis of two types of positive-sense RNAs (mRNA and cRNA) by utilizing its negative-sense viral RNA as a template. Following transcription and processing, viral mRNAs are transported to the cytoplasm for the purpose of translation (30). Complementary RNA acts as a template for the production of additional viral RNA. Beneath the plasma membrane, the vRNPs, in conjunction with HA, NA, M1 and M2, are assembled into viral particles (31). The NA protein facilitates the release of progeny viruses from infected cells by cleaving the glycosidic bonds between sialic acid receptors and sugar moieties, thereby enabling the infection of adjacent cells (32). Consequently, research efforts are underway to develop vaccines that target proteins integral to the influenza replication cycle.

## 3 The antigens utilized in H1N1 protein subunit vaccines

Within the influenza A virus genome, which encoded eleven proteins, the proteins that have been most extensively researched as potential vaccine antigen targets are HA, NA, the extracellular domain of the M2 protein (M2e), and NP. These proteins exhibit certain common features, including relatively high expression levels and conserved epitopes.

### 3.1 Hemagglutinin (HA)

HA head comprises several immunodominant antigenic sites (33). Varma et al. conducted a study utilizing a nasal gel formulated with recombinant computationally optimized broadly reactive HA antigen and cyclic guanosine monophosphate-adenosine monophosphate for the administration of the influenza vaccine. The experimental findings indicated that the administration of the gel resulted in significantly elevated levels of IgG2c and IgA in the mice (34). This vaccine formulation elicited stronger serum humoral responses compared to the absence of the gel, while also inducing a mucosal IgA response.

HA stem possesses epitopes capable of eliciting cross-reactive antibody responses and exhibits a greater degree of conservation compared to the head domain (35). In a study conducted by Tsybalova et al., the bacterial flagellin gene was fused with a fragment of HA2 gene and subsequently expressed it in the Escherichia coli (E. coli), resulting in the creation of three recombinant proteins: Flag-4M2e, Flag-HA2-2-4M2e, and Flag-HA2-1-4M2e. Notably, a portion of the HA2 in Flag-HA2-1-4M2e is derived from the A/H1N1pdm09 strain. The study reported the presence of specific anti-M2e IgG and IgA antibodies in the serum of immunized mice, alongside the generation of anti-HA antibodies to targeting various subtypes of the influenza A virus (36).

**Figure 3:**
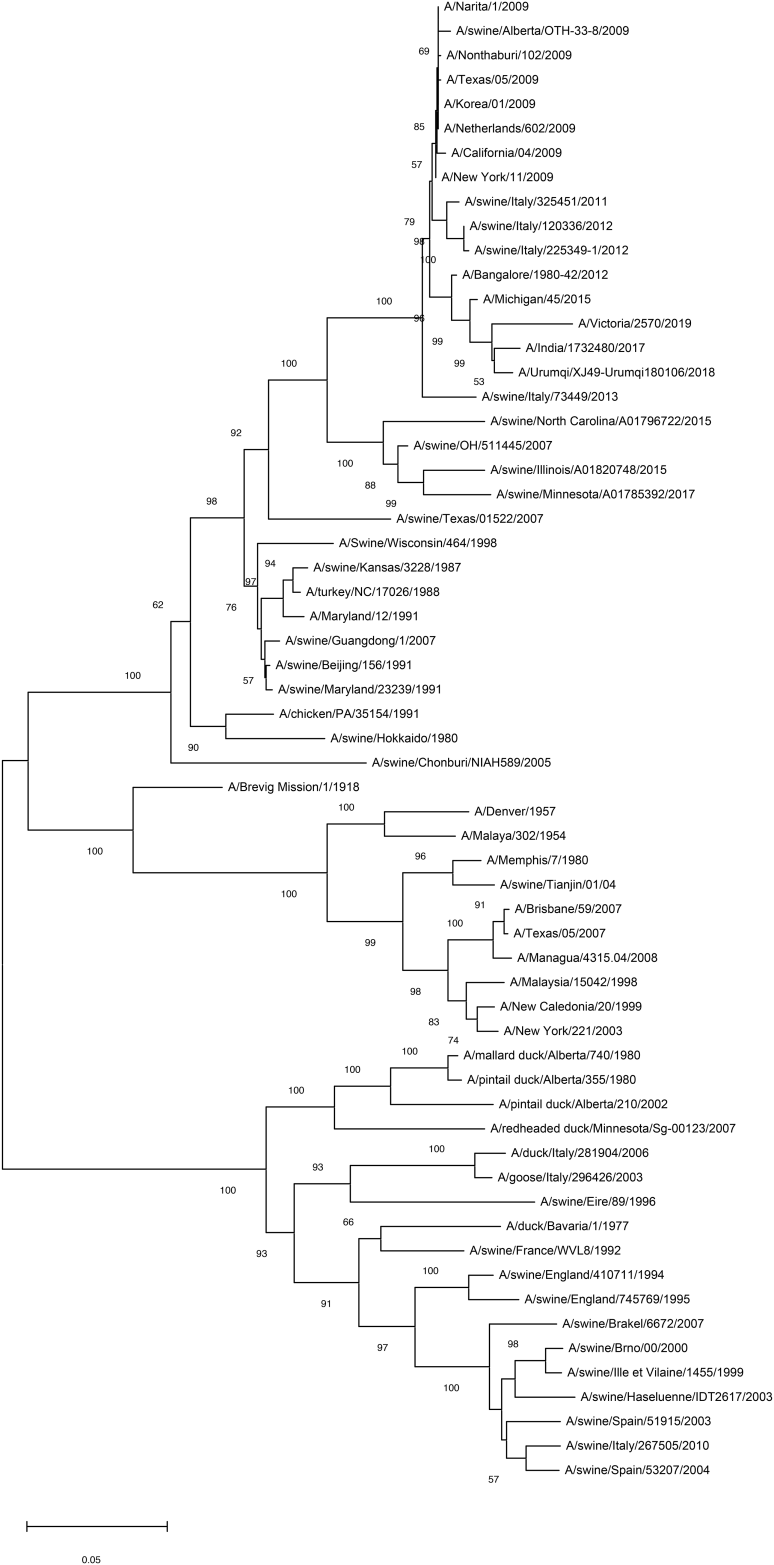
Mapping of antigenic clusters on phylogenetic tree of the HA region of the H1N1 HA nucleotide sequences. By analyzing the phylogenetic tree of the HA region of H1N1 virus, common mutations and conserved sequences that are common in different virus strains can be identified. These shared mutations and conserved sequences may serve as potential targets for the development of broad-spectrum vaccines, given their presence in multiple strains of the virus, thereby potentially offering a more extensive protective effect. Scale bar indicates nucleotide substitutions per site.

H1N1 acts as a multihoming virus, evolving through antigenic drift and shift as it spreads across species. Antigenic drift occurs due to point mutations in surface antigens, such as HA and NA, during viral replication. HA, a primary target for influenza vaccines, is central to understanding influenza’s sequence evolution (**Figure 3**). Recent advancements in H1N1 HA-based vaccine design have emphasized strategies like immune focusing, nanoparticle-based designs, and chimeric HA constructs to enhance cross-protection against diverse influenza strains (2). This focus is particularly pertinent in the context of viral antigenic drift and shift. Immune focusing redirects the immune response toward conserved regions, particularly the stem, which mutates less frequently (37). Techniques to achieve this include removing or masking the HA head to expose the conserved stem and developing HA mini-stem vaccines that use only the stem region. Preclinical studies show these approaches can induce broadly neutralizing antibodies (bnAbs) effective across multiple influenza subtypes (38). Nanoparticle vaccines also show promise by presenting conserved HA epitopes in structured arrays that better mimic the viral structure, leading to stronger immune responses. Chimeric HA vaccines replace the variable HA head with non-relevant sequences, guiding the immune system to focus on the stem and enhancing cross-reactivity among H1N1 strains (39). For instance, McCraw et al. created an HA stem-based chimeric nanoparticle vaccine using helix-A sequences on a hepatitis B virus capsid scaffold, producing cross-reactive antibodies that provided protection in mice (40). Mosaic HA vaccines further expand immune coverage by integrating epitopes from multiple strains. Together, these innovations offer potential pathways toward more universal and durable influenza vaccines (41).

Recent research has identified novel neutralizing antibodies that specifically target stem domain epitopes within the pre-fusion conformation of the HA protein, which are not accessible in the post-fusion conformation (42). In another study, McMillan et al. introduced a molecular clamp that effectively stabilized the formation of a stable HA trimer in its pre-fusion state. In the context of the H1N1 pmd09 challenge, C57BL/6 mice that received this treatment in conjunction with an adjuvant exhibited no significant weight loss or mortality, and cross-protection was successfully elicited in ferrets. Furthermore, vaccines stabilized by the HA clamp also generated neutralizing serum antibodies (43). These findings suggest that this innovative platform holds significant potential for the development of H1N1 vaccines, particularly in addressing the challenges posed by high mutation rates in the HA head and the limited efficacy of the HA stem.

### 3.2 Neuraminidase (NA)

NA has the activity of hydrolyzing sialic acid, which helps the virus to be released from the host cell. Numerous studies have indicated that the levels of both anti-NA and anti-HA antibodies diminish over time; however, experimental findings in murine models suggest that the decline of anti-NA antibodies occurs at a slower rate than that of anti-HA antibodies (44).

Kim et al. conducted a study on the 2009 pandemic H1N1 NA influenza virus-like particles (VLPs) vaccine. Their research involved animal models, which revealed that mice immunized with the VLP vaccine exhibited a cross-protective immune response when compared to those immunized with inactivated split vaccine. Notably, this immune response was unaffected in mice deficient in the Fc receptor γ-chain (45). Deng et al. demonstrated the expression of NA tetramers and monomers using Expi293F cells, revealing that only the tetrameric form of NA exhibited enzymatic activity. This tetrameric NA provided enhanced protection to mice against viral infection and elicited a greater production of specific binding antibodies (46). The active site of the NA enzyme is situated within its head domain (47). Furthermore, there exists potential for the incorporation of additional subunits within the NA head domain to augment its immunogenicity, or for the combination of NA with other antigens to develop a multi-component vaccine targeting various infectious diseases.

### 3.3 The extracellular domain of the M2 protein (M2e)

The matrix protein is composed of two components, M1 and M2. The M2 serves as a proton channel, playing a crucial role in regulating pH levels during the processes of viral entry and replication within host cells (48). The M2e is distributed across the surface of the viral particles; however, it is obscured by the HA and NA antigens. This structural arrangement results in M2e being less accessible to antibodies specific to influenza, thereby reducing its susceptibility to antigenic variation and contributing to its greater conservation (49).

The immune response elicited against the M2e epitope is characterized by its relative weakness, and the resulting specific antibodies exhibit an inability to neutralize the virus effectively. Consequently, the incorporation of carriers or adjuvants is crucial for enhancing the efficacy of M2e-based vaccines (2). In a particular study, researchers successfully expressed a fusion protein designated Flg4M2eHA2-1, which comprises two copies of the human consensus M2e sequence (M2eh) and two copies of the M2e sequence from the A/H1N1pdm09 strain (M2es), all linked to the C-terminus of the S. typhimurium flagellin protein FljB. Animal studies demonstrated that mice immunized with the recombinant protein exhibited high titers of M2e-specific IgG antibodies in both serum and bronchoalveolar lavage fluid; however, the immune response to the HA2 component was notably weak (50). This diminished response may be attributed to the positioning of the HA2 sequence between the flagellin and the tandem M2e copies.

### 3.4 Nucleoprotein (NP)

NP is a prevalent intracellular protein that plays a critical role in the transcription and replication of the viral genome, thereby affecting the host specificity and virulence of viruses (51). Upon infection with an influenza virus, NP serves as the primary antigen identified by host cytotoxic T lymphocytes, ultimately contributing to the eradication of the virus (52).

In a recent study, trimethyl chitosan was utilized both as a mucosal adjuvant and as a delivery system to create nanoparticles incorporating purified HA2 and NP proteins, with a focus on assessing their immunogenicity. *In vitro* analyses conducted on primary human nasal epithelial cells demonstrated that trimethyl chitosan nanoparticles (TMC nPs) enhance the uptake of HA2 and NP proteins. When compared to control TMC nPs devoid of active components, human nasal epithelial cells treated with various formulations of influenza nanoparticles (including HA2-TMC nPs, NP-TMC nPs, and HA2-NP-TMC nPs) exhibited a significantly elevated production of pro-inflammatory cytokines, suggesting a heightened immune response. Furthermore, in the subsequent experiments simulating viral infection, the influenza nanoparticle formulations were found to markedly inhibit the replication of the influenza virus (53).

## 4 The expression system utilized in the formulation of H1N1 protein subunit vaccines

The choice of recombinant protein expression system is a critical step in the formulation of recombinant protein subunit influenza vaccines. The protein has been produced in various heterologous host cells, including E. coli, yeast, insect cells and mammalian cells (54). Each of these systems possesses distinct characteristics that influence their suitability for specific applications.

### 4.1 E. coli

The E. coli expression system is widely recognized as a prevalent platform for the expression of recombinant proteins (55). The expression system utilizing E. coli has gained widespread acceptance as a host for the production of recombinant heterologous proteins, owing to its advantages, which include high expression levels, rapid production cycles, and cost-effectiveness.

Two different HA fragments (rHA1_1-326_ and rHA1_53-269_) were expressed in E. coli and used to vaccinate mice, where rHA1_1-326_ demonstrated a significant level of protection against a homologous H1N1 viral challenge. In contrast, the rHA1_53-269_, which encompasses neutralizing epitopes, fails to form higher-order oligomers. Therefore, it does not elicit the same protective response as rHA_1-136_, which contains both neutralizing epitopes and a trimerized domain (56). It is well established that E. coli, being a prokaryotic organism, may result in inadequate post-translational modifications, the formation of inclusion bodies, and potential protein degradation (57). Molecular chaperones play a crucial role in assisting newly synthesized, aggregated or misfolded proteins in achieving their native conformations, and their application has been extensively documented to enhance the expression of various proteins (58). One particular study highlighted the use of heterologous caveolin-1 as a chaperone, in conjunction with the Oct1 DNA-binding domain as a fusion partner, which significantly improved the solubility of influenza HA (59).

### 4.2 Yeast

The yeast expression system possesses several advantageous characteristics, including the capacity to elicit specific immune responses against the recombinant antigens, its adjuvant properties (60) and its role in the activation of the antigen presenting cells (APCs) (61). Notable yeasts utilized in this context include Saccharomyces cerevisiae and Pichia pastoris, among others. Saccharomyces cerevisiae is the most frequently employed yeast for the production of recombinant proteins (62). However, Pichia. Pastoris is increasingly favored over Saccharomyces cerevisiae due to its ability to achieve high cell density cultures, which is facilitated by its aerobic respiration and the expression of proteins under the tightly regulated methanol inducible AOX1 promoter (63).

Kopera et al. conducted a study examining the potential of the Pichia cells for the production of a soluble H1N1 HA antigen. Their immunization experiments revealed that the H1 antigen elicited a robust HI-immune response in murine models (64). Concurrently, research is underway to develop yeast-based oral influenza vaccines. Oral vaccination has the capacity to activate both the humoral and cellular immune responses at systemic and mucosal levels, thereby representing an effective strategy for inducing protective sIgA responses (65). Lei and colleagues utilized Saccharomyces cerevisiae EBY100 to successfully formulate oral vaccines for H7N9-HA (66) and H5N1-HA (67). Subsequent animal studies demonstrated significantly elevated levels of HA-specific antibodies and HI titers were detected, which are anticipated to confer protection against both homologous and heterologous strains of influenza viruses. These findings are promising for the development of an oral vaccine targeting H1N1 using yeast expression systems.

### 4.3 Insect cells

The baculovirus expression vector system has emerged as a critical platform for the production of recombinant proteins utilized in vaccines (68). The baculovirus expression system is characterized by several key features: 1) a high safety profile, as it is unable to complete a full replication within mammalian cells; 2) a substantial capacity for the incorporation of exogenous genes; and 3) an efficient expression of these genes.

In a research investigation, H1N1 HA gene was successfully cloned into baculovirus expression vector. The recombinant vector was subsequently introduced into E. coli for propagation, followed by transfection into SF9 insect cells for protein expression. Comparative analyses using hemagglutination inhibition and enzyme-linked immunosorbent assay demonstrated that the recombinant H1N1 protein, when formulated with adjuvant, elicited a greater antibody response against viral challenge than the commercially available H1N1 influenza vaccine. Furthermore, the results from animal studies indicated that the protective efficacy of the novel vaccine was 96%, surpassing that of existing alternatives (69).

### 4.4 Mammalian cells

Mammalian cell expression systems are increasingly favored for H1N1 antigen production in influenza vaccine development due to several advantages over traditional egg-based systems (70). Cell-based influenza vaccines are particularly advantageous as they help avoid the antigen mismatch issues that can arise with chick embryo-based production (71). Common mammalian cell lines for recombinant protein production include Chinese hamster ovary (CHO) cells and human embryonic kidney 293 (HEK293) cells (72, 73). CHO cells, which are non-secreting, rarely produce endogenous proteins, making it easier to separate and purify the target protein after expression. Additionally, CHO cells can yield high protein quantities in large-scale cultures, though their glycosylation patterns differ from those in humans (74). HEK293 cells, on the other hand, are easy to culture, have high transfection efficiency, and can produce recombinant proteins in substantial quantities, making them suitable for transient transfection. Derived from human cells, HEK293 cells can better simulate human glycosylation patterns, yielding proteins more closely matching human conformation (75). However, their longer doubling time in suspension culture can reduce production rates compared to CHO cells (76). Overall, these mammalian systems are widely used for producing recombinant subunit proteins in vaccine development.

In a research investigation, the modified HA was produced as a soluble trimeric protein within mammalian cell systems. Subsequent assessments indicated that the recombinant HA exhibited functional activities comparable to those of their native counterparts present on the surface of influenza viral particles (77). However, the researchers did not assess the immunogenicity of the expressed HA proteins. In a separate investigation, the researchers developed chimeric HA and employed chimpanzee adenoviral vectors (AdC68) vaccine platform to produce recombinant adenoviral plasmids. Then they expressed the chimeric HA protein in HEK293 cells. Analysis of the experimental outcomes revealed that the AdC68-cH1-H7 construct demonstrated significant immunogenicity in murine models and conferred protection against challenges with various strains of the influenza virus (39). The suboptimal efficacy observed in other chimeric HA vaccines may be attributed to the concealment of antigenic sites resulting from their conformational structures.

## 5 Adjuvant utilized in H1N1 protein subunit vaccines

Adjuvants are incorporated into or administered alongside the vaccine antigens to strengthen the immune response, particularly useful in addressing antigenic mismatches between vaccine strains and circulating pathogens (78). During the 2009 H1N1 pandemic, adjuvants played a critical role in improving vaccine efficacy, especially for high-risk groups. Traditional adjuvants, such as aluminum salts, MF59, and AS03, were commonly used (**Table 3**), while technological advancements have led to the development of numerous new adjuvants (**Table 4**). Based on their mechanisms, adjuvants can be classified into two main types: immunostimulants, which activate and mature antigen-presenting cells (APCs), and delivery systems, which improve antigen uptake and presentation by APCs (93).

**Table 3:** Licensed influenza vaccine adjuvants for human use.

Adjuvant name	Adjuvant type	Formulation	Licensed (year)	Description
Alum	Aluminum as mineral salt	Aluminum hydroxide/ Aluminum phosphate/ Potassium aluminum sulfate	1926	Delays antigen release; Increases antigen phagocytosis; Enhances antigen presentation
MF59	Oil-in-water emulsion	Squalene, polysorbate 80, sorbitan trioleate	1997	Increases antigen phagocytosis; Enhances antigen presentation; Activates the inflammasome
Virosome	Liposome	Lipids, hemagglutinin	2000	Enhances antigen presentation; Interacts with B cells leading to T-cell activation
AS03	Oil-in-water emulsion	α- tocopherol, squalene, polysorbate 80	2009	Delays antigen release; Induces the production of cytokines and recruitment
AF03	Oil-in-water emulsion	Squalene, polyoxyethylene cetostearyl ether, mannitol, sorbitan oleate	2011	Enhances antigen presentation; Activates the inflammasome

**Table 4:** New influenza vaccine adjuvants under study.

Target/Type	Adjuvant name	Structure	Description	Reference
TLR-ligands	Poly I: C	Double-stranded RNA polyriboinosinic polyribocytidylic acid	Recognizes TLR3; Induces TRIF dependent signaling pathway; Promotes the release of proinflammatory factors	(79–81)
	Flagellin	A protein composed of 494 amino acids	Recognizes TLR5; Promotes the production of cytokines and chemokines	(82, 83)
	β-glucan particle	A polysaccharide compound in which glucose molecules are connected by glucoside bonds	Recognizes TLR1/2, TLR2/6; Induces stronger cytokine secretion	(84)
	Mannan	Mannose	Recognizes TLR2; Promotes the production of IL-1, IL-6 and TNF	(85, 86)
	Outer Membrane Vesicle (OMV)	LPS, Peptidoglycans, Phospholipids, Proteins	Recognizes TLR2, TLR4; Stimulates the immune innate system; Promotes APCs activation and enhances antigen presentation	(87)
Nanoparticles	Curdlan-chitosan conjugate nanoparticle	Curdlan; Chitosan	Stimulates the activation of APCs; Enhances mucosal immunity	(88)
	Selenium nanoparticle	Mineral selenium or Organic selenium	Enhances immune responses; Reduces oxidative stress; Targets macrophages;	(89)
	PLGA	PLA; PGA	Enhances antigen delivery; Prolongs antigen release time; Induces of IL-6, IL-12	(90, 91)
	Noble metallic nanoparticle	Gold/ Silver	Enhances antigen presentation; Promotes cytokine release; Prolongs antigen release	(92)

### 5.1 Immunostimulant adjuvants

**Figure 4:**
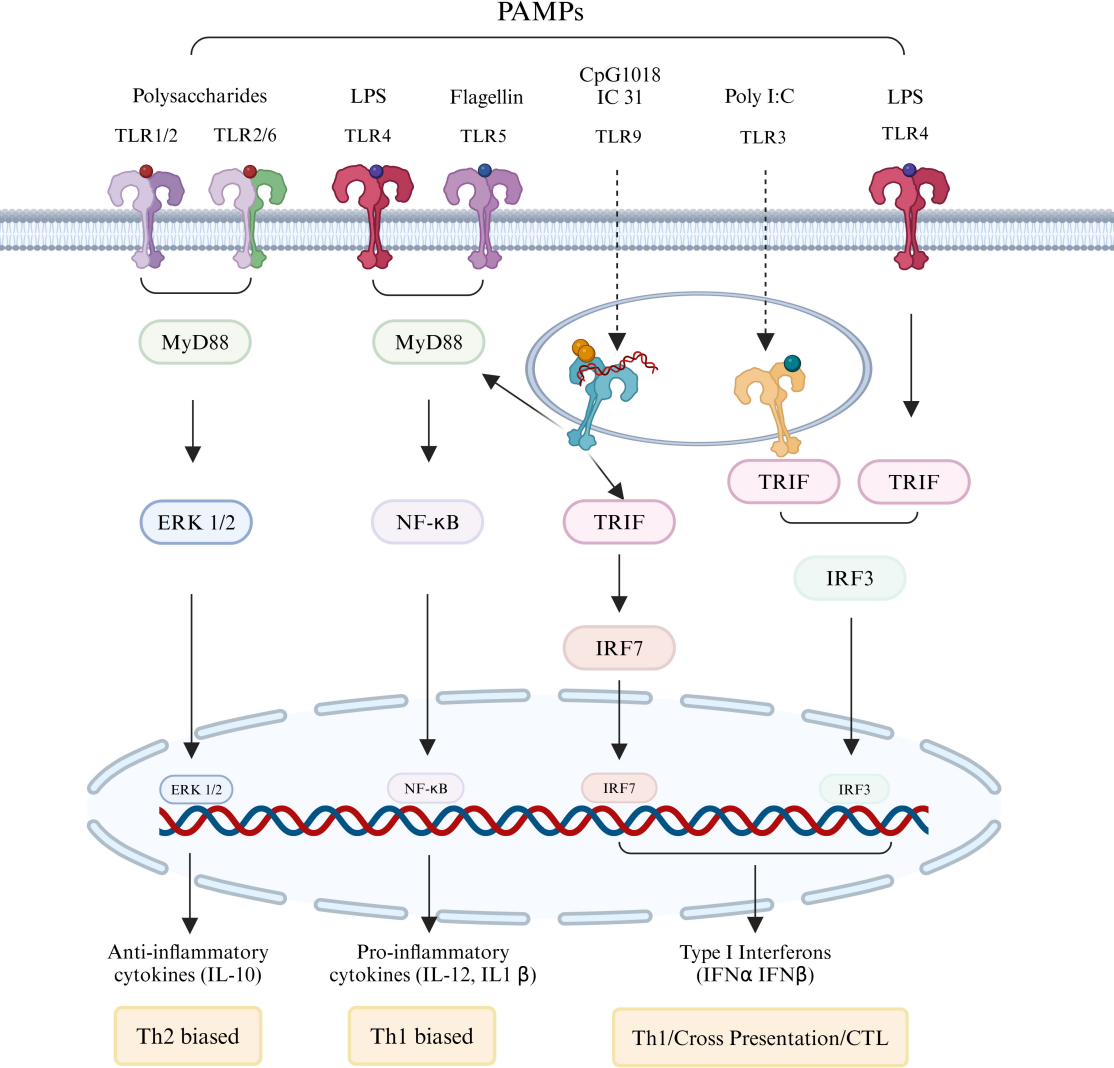
Immunostimulants regulating adaptive immune responses by activating TLRs. Membrane-associated TLRs 1, 2, 4, 5, and 6, as well as endosomal membrane-type TLRs 3 and 9, form either homologous or heterodimeric complexes upon interaction with specific pathogen-associated molecular patterns (PAMPs). With the exception of TLR3, which operates through the TRIF signaling pathway, the majority of these receptors activate downstream immune responses via the MyD88 pathway. The engagement of PAMPs leads to the enhancement of antigen presentation by stimulating the corresponding TLRs and their associated signaling pathways, resulting in the production of interferons, cytokines, and chemokines, thereby ultimately bolstering the adaptive immune response.

TLRs play a crucial role in the activation of innate immunity through their interaction with pathogen-associated molecular patterns (94). Additionally, TLR adaptor molecules serve as a link between innate and adaptive immune responses (95). The activation of intracellular TLRs generally triggers a robust immune response characterized by the production of type I interferons (IFNs) and inflammatory cytokines (96). Consequently, the incorporation of targeted TLR adjuvants into protein subunit influenza vaccine has the potential to enhance their immunogenicity (**Figure 4**).

#### 5.1.1 TLR4 agonists

TLR4 facilitates the production of various proinflammatory mediators through both MyD88-dependent and MyD88-independent pathways, engaging the rapid and slow transcription factors nuclear factor κB and mitogen-activated protein kinase, respectively (97). The Bacterial Enzymatic Combinatorial Chemistry (BECC) platform has yielded two novel lipid A-based TLR4 agonist candidates, designated BECC438 and BECC470 (98). Research conducted by Haupt et al. has characterized two innovative BECC adjuvants, which, when combined with a recombinant influenza HA protein, demonstrated the ability to provoke a more robust and extensive immune response compared to traditional adjuvants such as alhydrogel and phosphorylated hexa-acyl disaccharide. This enhanced immune response was observed in both homologous and heterologous challenges. Notably, this superior efficacy was achieved with more than seven times less antigen and ten times less adjuvant. However, it is important to highlight that in aged mice, only the vaccine adjuvanted with BECC470 elicited a strong response in H1-specific IgG (99). In a novel methodology, some researchers synthesized Lipo-(1V270 + 2B182C) through the co-encapsulation of the TLR7 agonist 1V270 and the TLR4 agonist derivative 2B182C within liposomes. The H1N1 vaccine that incorporates the combined adjuvant was assessed for its ability to augment the production of anti-HA and anti-NA antibodies, as well as to enhance the cross-reactivity of sera specific to HA and NA (100). Therefore, the application of a combined adjuvant represents a promising strategy for enhancing the immunogenicity of influenza protein subunit vaccines.

#### 5.1.2 TLR9 agonists

TLR9 plays a critical role in the immune response to microbial unmethylated CpG DNA and is activated by synthetic CpG-oligodeoxynucleotides (CpG-ODNs) (101). Generally, CpG-ODNs facilitate the maturation of dendritic cells into professional APCs, stimulate Th1 responses that promote the production of IFN-γ and CD8+ T cells, and enhance antibody responses, thereby contributing to protective immunity (102). B type CpG-ODNs are commonly utilized as a vaccine adjuvants in human applications (103), as they elicit strong innate immune responses through a TLR9-dependent NF-κB signaling pathway (104). Research conducted by Li et al. demonstrated that CpG1018 significantly improved the cytotoxic T lymphocytes response and humoral immune response in murine models when compared to the MF59-mimicked AddVax (105). Furthermore, studies indicate that lipid nanoparticles conjugated with CpG enhance both the adjuvant efficacy and safety profile of CpG-ODN (106, 107). Consequently, it has been incorporated into routine studies of influenza split vaccines, thereby expanding their protective efficacy against the influenza virus (107). As discussed above, this approach represents a promising strategy for enhancing the safety and effectiveness of protein subunit influenza vaccines.

### 5.2 Delivery system adjuvants

#### 5.2.1 Emulsion delivery adjuvants

Conventional adjuvants, such as alum, exhibit immunogenicity levels comparable to those of unadjuvanted vaccines. Consequently, there has been an investigation into alternative adjuvants, including oil-in-water formulations. Notably, AS03 and MF59 are oil-in-water adjuvants that are frequently utilized in influenza vaccines (108).

##### 5.2.1.1 AS03

AS03 has been shown to enhance both antibody and T-cell responses to HA present in the split antigen. The immune-boosting characteristics of AS03 also lead to a significant antigen-sparing effect, which is particularly important in light of the limited global capacity for pandemic influenza antigen production (109). However, an increased incidence of narcolepsy type 1 has been reported in several European nations (110), with this risk being specifically associated with the Pandemrix influenza vaccine that incorporates AS03 (111). There exists a divergence of opinions regarding the pathogenesis of narcolepsy type 1 linked to H1N1 infection and the Pandemrix vaccine. Some studies propose that the disease may be a result of cross-reactivity between autoreactive CD4^+^T cells and H1N1 influenza antigens (110, 112, 113), while other research indicates a lack of such cross-reactivity (114, 115).

##### 5.2.1.2 MF59

The MF59 adjuvant has been demonstrated to elicit proinflammatory chemokines and cytokines in both murine and human models, thereby facilitating enhanced antigen uptake and improving the processing and presentation of antigens (116). An influenza vaccine formulated with MF59 adjuvant has been shown to elicit a more robust and prolonged antigen-specific immune response in comparison to vaccines lacking adjuvants (117). Furthermore, a recombinant influenza vaccine utilizing rHA in conjunction with the MF59 adjuvant, produced in SF9 cells via a baculovirus expression system, yielded higher HI antibody titers than those observed with alum-adjuvanted vaccines (118). Additionally, a similar H1N1 HA vaccine combined with MF59 adjuvant demonstrated a significant Th1 immune response and cytotoxic T lymphocytes activity in BALA/c mice (119). MF59 is recognized for its favorable safety profile and efficacy, leading to its widespread application. AddaVax™ serves as a preclinical grade equivalent of MF59. In a recent investigation, AboulFotouh et al. successfully converted liquid AddaVax™ recombinant HA influenza vaccines into dry powders utilizing thin-film freeze-drying technology. Evaluation in animal models indicated that the immunogenicity of the vaccine was preserved following this conversion (120).

#### 5.2.2 Nanoparticles delivery system

**Figure 5:**
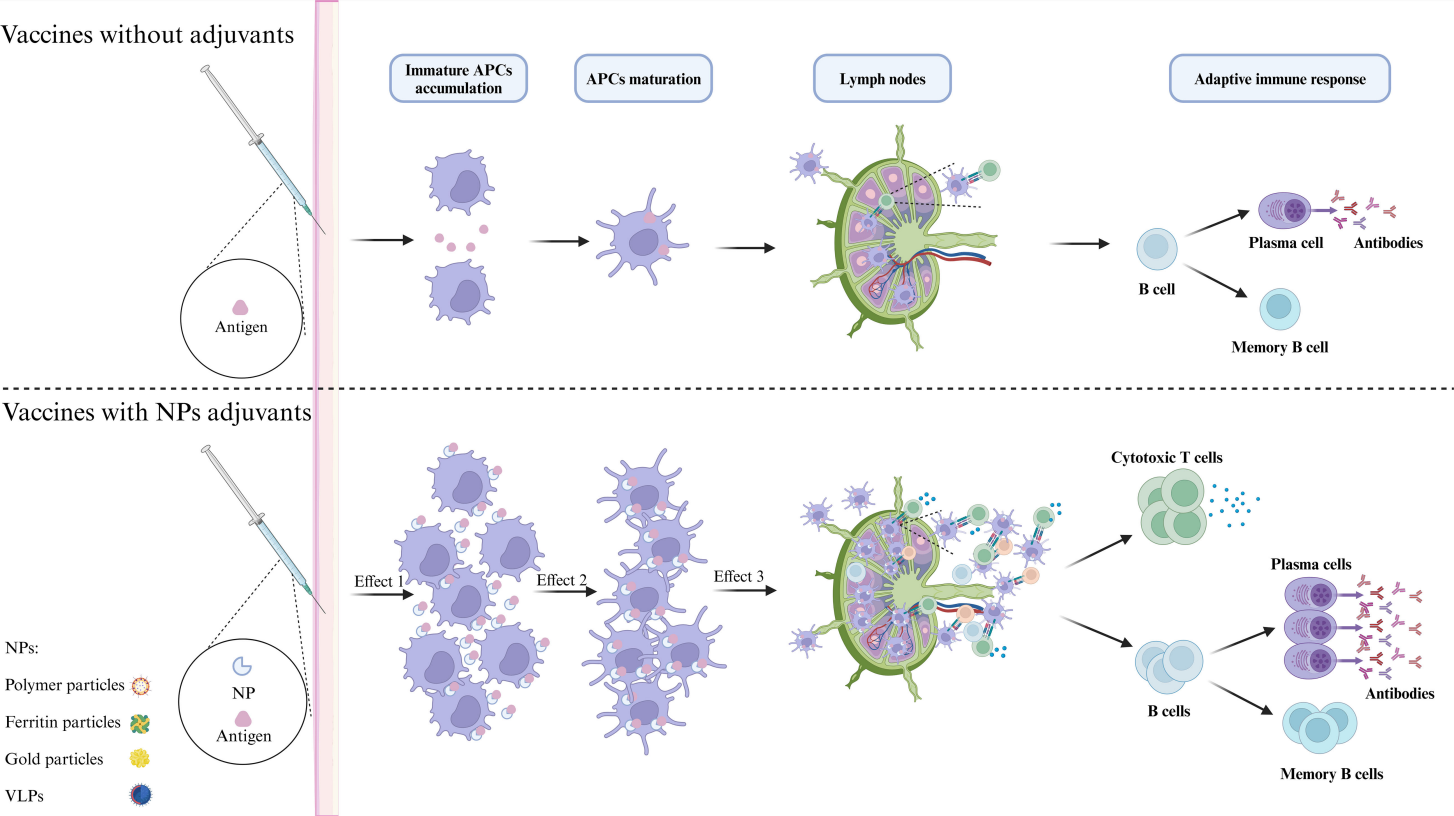
Molecular mechanism of NPs enhancing immune response. Antigens can be incorporated on the surface or within nanoparticles (NPs) through various methods, which may enhance their stability, prolong antigen half-life, and improve delivery efficiency. The primary mechanism of nanoparticle adjuvants is to facilitate antigen presentation. First, nanoparticle adjuvants form an antigen depot that protects the antigens and enables gradual release, attracting and activating additional APCs to the injection site. Second, by targeting APCs, nanoparticle adjuvants sustain stimulation, increasing antigen uptake and presentation by mature APCs and enhancing MHC-antigenic peptide signaling. Finally, these adjuvants facilitate the transport of antigens to lymph nodes, which are critical locations for the initiation of immune responses. Within these nodes, processed peptides are presented to T cells, thereby effectively activating an adaptive immune response.

Nanoparticle-based vaccine platforms have significantly transformed the development of influenza vaccines by enhancing immune responses and providing extensive protection against a range of viral strains, including H1N1 (121). These platforms more closely mimic the virus structure than traditional vaccines, resulting in enhanced antigen presentation and stronger immune responses (**Figure 5**). For H1N1 and other influenza vaccines, nanoparticle platforms improve antigen presentation, induce broadly neutralizing antibodies (bnAbs), and increase stability. By presenting viral antigens in an organized manner that closely resembles the virus, nanoparticles enhance immune recognition, leading to stronger, longer-lasting responses (122). For example, ferritin-based HA stem vaccines elicit bnAbs that offer cross-protection against multiple influenza subtypes (123). Additionally, nanoparticles protect antigens from degradation, increasing stability and improving efficacy. The versatility of nanoparticles allows for their engineering to carry various antigens and adjuvants, enabling customization for different viral strains or components. Self-assembling protein nanoparticles, for instance, can incorporate multiple epitopes from different viral strains, thereby improving cross-protection and potentially reducing the need for frequent updates (124).

There are two main types of nanoparticle vaccines: those encapsulating antigens within nanoparticles, such as liposomes and polymer nanoparticles, and those displaying antigens on nanoparticle surfaces, like protein nanoparticles and virus-like particles (VLPs) (125). Lipid nanoparticles are now widely used to deliver nucleic acid vaccines, as seen in COVID-19 (126). While preparation and purification of some nanoparticles can drive up production costs and raise stability and biosafety concerns, self-assembled nanoparticles offer advantages in these areas (125). However, challenges such as complex production, scalability, and regulatory hurdles limit widespread adoption.

##### 5.2.2.1 Polymer-nanoparticles

Polymer nanoparticles demonstrate several advantageous properties, including biocompatibility, a high capacity for loading immune-related components, and solubility in water (127). Polymers can be categorized into two main types: natural polymers and synthetic polymers. Among these, chitosan and poly (lactic-co-glycolic acid) are the most extensively researched nanoparticles. Chitosan, as a cationic polysaccharide, is considered a highly promising pharmaceutical material due to its non-toxic nature, biodegradability, and superior biocompatibility (128). Furthermore, nanoparticles composed of chitosan and its derivatives, when loaded with antigens, have demonstrated the ability to elicit both cellular and humoral immune responses (129).

Sadeghi et al. developed a poly-epitope construct based on HA-NP-M2, which was combined with chitin or/and chitosan particles to serve as a mucosal adjuvant. They assessed the intranasal induced immune response elicited in murine models. The findings indicated that the group receiving 50ug of protein along with 100ug of chitosan exhibited a significantly elevated production of IgG2a antibodies and cytokines, as well as enhanced viral neutralization capabilities in experimental assays (130).

Polymer-nanoparticle hydrogels represent a category of supramolecular hydrogels where the polymeric constituents are held together by dynamic, multivalent noncovalent interactions involving both polymers and nanoparticles (131). The TLR7/8a-epitope nano-vaccine has been demonstrated to enhance local retention and improve drainage efficiency to the lymph nodes (132). In this context, Toy et al. conducted an experiment in which mice were immunized via intramural injections of the nanoparticles that were loaded with HA protein, in conjunction with TLR7-targeting R848 and RIG-I targeting PUUC adjuvants. The findings indicated that the nanoparticle-mediated delivery of the antigen, along with the dual adjuvant approach, significantly augmented the cellular immune response (133).

##### 5.2.2.2 Ferritin nanocages

Ferritin nanocages represent versatile nanocarriers that have been employed in the delivery, imaging and therapeutic administration of anticancer agents (134), while also facilitating novel approaches for vaccine delivery. Ferritin is composed of 24 protein subunits (135) that self-assemble into a shell-like structure exhibiting F432 symmetry (136). Ferritin exhibits exceptional physicochemical characteristics, encompassing a broad spectrum of pH resistance and significant thermal adaptability (137). The external surface of ferritin is capable of accommodating various antigens, facilitating their presentation in a polymeric form, which more accurately mimics the natural configuration of polymerized antigens (138).

Nie et al. developed a self-assembled nanoparticle vaccine, referred to as MHF, which is expressed in a soluble form in E. coli through the conjugation of multiple linear epitopes from HA2 and M2e to ferritin. Experimental data from animal studies indicate that mice immunized with the MHF vaccine generate a broad spectrum of neutralizing antibodies and exhibit a balanced Th1/Th2 immune response. Furthermore, these mice demonstrate a robust induction of IFN-γ-secreting lymphocytes and specific memory B cell responses (139). Subsequent investigations revealed that the serum of mice immunized intranasally with the MHF vaccine exhibited significantly higher neutralizing antibody titers and antibody-dependent cell-mediated cytotoxicity activity against H1N1 compared to those receiving the H3 split vaccine via subcutaneously administration (140). Consequently, the ferritin nanocage emerges as a promising platform for vaccine delivery aimed at enhancing mucosal immunity. Darricarrère et al. conducted a characterization of H1 HA-stabilized stem ferritin nanoparticle vaccines, specifically H1ssF and H3ssF, which were administered to Cynomolgus macaques either with the adjuvant AF03 or without it. The findings indicated that the group receiving the adjuvant exhibited the highest endpoint titer and demonstrated broad neutralizing responses against both H1 and H3, exhibiting a recognition mechanism similar to that of human broadly neutralizing antibodies (141). In this context, H1ssF was assessed in a Phase I clinical trial, where it was found to be both safe and well tolerated (142).

##### 5.2.2.3 Gold nanoparticles

(AuNPs) AuNPs consist of an outer layer of organic ligands surrounding a gold core (143). Their successful application in drug delivery has led to their consideration as an optimal vehicle for vaccine delivery. AuNPs have been shown to inhibit the binding of transfer RNA (tRNA) to ribosomes, decrease adenosine triphosphate (ATP) levels, and do not generate reactive oxygen species (ROS), which contributes to their safety profile (144). Additionally, AuNPs can facilitate the delivery of antigens to antigen-presenting cells, thereby activating the adaptive immune response and exhibiting adjuvant properties (145).

In one study, the M2e protein of the H1N1 virus was conjugated to the surface of AuNPs and characterized alongside soluble CPG as a freeze-dried vaccine formulation (AuNP-M2e + sCpG). This vaccine demonstrated favorable thermal stability. The assessment of the vaccine indicated that freeze-drying did not compromise its immunogenicity, providing protection to both mice and ferrets against influenza viruses (146). In a subsequent investigation, a microneedle patch vaccine (AuNP-M2e + sCpG) was developed using a similar approach. In murine models, 85.4% of the AuNPs were successfully delivered to the skin, and immunized mice exhibited elevated levels of IFN-γ, TNF-α, IL-6, and IL-7 (147). In another study, Kim et al. engineered porous gold nanoparticles that cleaved the disulfide bond of HA to inhibit membrane fusion and prevent viral entry into host cells, thereby obstructing viral infection (148).

##### 5.2.2.4 Virus-like particles (VLPs)

VLPs possess a hollow architecture that resembles the spatial configuration of natural virions, yet they lack viral nucleic acids. These particles are capable of directly engaging with antigen-presenting cells (149). Consequently, VLPs can activate the human immune system in a manner analogous to viral infection, thereby effectively eliciting immune protection.

Makarkov et al. developed a plant-derived VLP based on the H1 antigen, which can be internalized via various endocytic pathways. This characteristic allows for a broader range of antigen processing and presentation mechanisms compared to recombinant soluble H1 protein and the 2009 H1N1 monovalent split vaccine (150). In a separate investigation, Menne et al. utilized insect expression system to produce NA2 VLPs, which were administered to mice via intranasal and intramuscular routes. The results indicated that intramuscular vaccination conferred superior protection. Specifically, intramuscular administration of NA2 VLPs containing the NA2 antigen derived from A/Perth/16/2009 offered protection against a distantly related heterologous mouse adapted H3N2 virus. However, this approach did not provide protection in mice subjected to a lethal heterosubtypic challenge with the H1N1 virus (A/California/04/2009) (151).

## 6 Animal models utilized for the evaluation of H1N1 protein subunit vaccines

Mice and ferrets serve as the principal animal models for investigating human influenza infections, which are crucial for examining pathogenesis, assessing therapeutic interventions, and comprehending the mammalian adaptation needs of avian viruses (3, 152). Due to their evolutionary proximity to humans, nonhuman primates are typically favored as preclinical models for the evaluation of vaccines prior to their application in human clinical trials (141).

### 6.1 Mice

In the field of influenza research, mice serve as the predominant animal model, with the C57BL/6 (103, 153) and BALB/c (154–156) strains being the most frequently utilized among inbred mouse populations.

Ong et al. employed the NvC-M2ex3 construct to immunize mice subcutaneously following a prime-double boost regimen. Subsequently, the mice were subjected to intranasal infection with mouse-adapted strains of H1N1 or H3N2. The results indicated no significant weight loss or observable signs of illness in the vaccinated mice, who exhibited elevated levels of IFN-γ and IL-12 in the pulmonary tissue following challenges with H1N1 and H3N2 (157). Additionally, AdC68 was utilized to generate recombinant chimeric HA, which were engineered by amalgamating multiple head domains from various influenza virus subtypes onto a single stalk domain. The AdC68-cH1-H7 construct demonstrated a significant reduction in viral infection and conferred protection to mice against lethal challenges posed by both H1 and H7 subtypes (39). However, it is important to note that mice are not natural hosts for the influenza virus. Unlike humans, mice exhibit replication of influenza A virus primarily in the lower respiratory tract, and research has indicated that the pathogenesis of the influenza virus in murine models differs to some extent from that observed in humans (158).

### 6.2 Ferrets

Ferrets were the inaugural animal species subjected to experimental infection with the influenza virus (152) and are regarded as the ‘gold standard’ for evaluating the transmissibility of influenza viruses through respiratory droplets (3). They exhibit a higher susceptibility to human influenza viruses compared to mice, possess a distribution of sialic acid residues in the respiratory tract that closely resembles that of humans, and demonstrate a comparable progression of disease and immune responses (159).

In a research investigation, partial female Fitch ferrets underwent preimmunization followed by intramuscular administration of 15 ug of computationally optimized broadly reactive NA antigen within a prime-boost framework. Subsequent challenges with influenza viruses H1N1 and H5N1 demonstrated that the vaccine successfully induced the production of specific neutralizing antibodies and effectively curtailed viral transmission among the ferrets (160).

### 6.3 Nonhuman primates

As research has advanced, nonhuman primates have increasingly been utilized in biomedical studies (161). Among these, the cynomolgus monkey and the rhesus monkey have emerged as the most frequently employed species in this field (162). The genetic and physiological similarities of primates to humans, coupled with their extended lifespan, facilitate long-term studies (163). Research indicates that rhesus monkeys exhibit a greater propensity to display clinical symptoms when infected with H1N1, while cynomolgus monkeys demonstrate elevated levels of viral replication and increased body temperature in both the upper respiratory tract and lungs (164).

In a study, Swart et al. developed a subunit vaccine targeting the H1 stem protein, incorporating two additional point mutations to enhance the protein stability. They subsequently characterized the vaccine’s formulation efficacy in conjunction with various adjuvants in both murine and cynomolgus monkey models. A sustained humoral immune response specific to HA was observed in cynomolgus monkeys that were administered different adjuvants containing the H1 stem protein (165).

Typically, murine models are predominantly utilized in the initial phases of influenza vaccine research, while ferret models are employed in the intermediate stages. Ultimately, non-human primate models are introduced for a more comprehensive evaluation, contingent upon specific laboratory conditions and other relevant factors.

## 7 Conclusion

Protein subunit vaccines constitute a promising and adaptable strategy for addressing the A/H1N1pdm09 influenza virus. Recent advancements in antigen design, adjuvant technology, and delivery mechanisms have markedly improved the effectiveness of these vaccines, thereby establishing them as a crucial asset in the worldwide initiative to prevent influenza. HA remains the predominant antigen utilized in influenza vaccines; however, there is a growing body of research focused on alternative antigens associated with influenza viruses, such as M2e and NP. Additionally, vaccines based on NA-derived VLPs are currently under active investigation. There is also a trend toward the development of chimeric or combination vaccines, which involve the fusion of the HA stem with other proteins from influenza viruses or subunits from different viral proteins. Mammalian cell expression systems offer a promising alternative to traditional egg-based methods for H1N1 antigen production, with the potential for faster, scalable, and more accurate vaccine production. Nanoparticles derived from ferritin, liposomes, and VLPs have demonstrated significant potential in the effective delivery of influenza antigens, eliciting strong immune responses and offering cross-protection. Ongoing research aimed at overcoming the challenges posed by antigenic variability and the necessity for extensive, long-lasting protection positions protein subunit vaccines as integral components in forthcoming influenza prevention strategies, including the creation of more broadly protective universal influenza vaccines, enhancements in adjuvant and delivery system technologies, and the establishment of rapid response frameworks to effectively address emerging influenza viruses.
